# A Microbiological, Toxicological, and Biochemical Study of the Effects of Fucoxanthin, a Marine Carotenoid, on *Mycobacterium tuberculosis* and the Enzymes Implicated in Its Cell Wall: A Link Between Mycobacterial Infection and Autoimmune Diseases

**DOI:** 10.3390/md17110641

**Published:** 2019-11-14

**Authors:** Miroslava Šudomová, Mohammad Ali Shariati, Javier Echeverría, Ioana Berindan-Neagoe, Seyed Mohammad Nabavi, Sherif T. S. Hassan

**Affiliations:** 1 Museum of Literature in Moravia, Klášter 1, 664 61 Rajhrad, Czech Republic; sudomova@post.cz; 2 Kazakh Research Institute of Processing and Food Industry (Semey Branch), Semey 071410, Kazakhstan; shariatymohammadali@gmail.com; 3 Departamento de Ciencias del Ambiente, Facultad de Química y Biología, Universidad de Santiago de Chile, Casilla 40, Correo 33, Santiago 9170022, Chile; javier.echeverriam@usach.cl; 4 Research Center for Functional Genomics, Biomedicine and Translational Medicine, University of Medicine and Pharmacy “Iuliu-Hatieganu”, 400337 Cluj-Napoca, Romania; ioananeagoe29@gmail.com; 5 MedFuture Research Center for Advanced Medicine, University of Medicine and Pharmacy “Iuliu-Hatieganu”, 400349 Cluj-Napoca, Romania; 6 Department of Functional Genomics and Experimental Pathology, The Oncology Institute “Prof. Dr. Ion Chiricuţă”, 400015 Cluj-Napoca, Romania; 7 Applied Biotechnology Research Center, Baqiyatallah University of Medical Sciences, Tehran 14359-16471, Iran; 8 Department of Natural Drugs, Faculty of Pharmacy, University of Veterinary and Pharmaceutical Sciences Brno, Palackého tř. 1946/1, 612 42 Brno, Czech Republic

**Keywords:** *Mycobacterium tuberculosis*, autoimmunity, fucoxanthin, marine carotenoid, UDP-galactopyranose mutase, arylamine-*N*-acetyltransferase, pathogenesis

## Abstract

This study explored the antitubercular properties of fucoxanthin, a marine carotenoid, against clinical isolates of *Mycobacterium tuberculosis* (Mtb). Two vital enzymes involved in Mtb cell wall biosynthesis, UDP-galactopyranose mutase (UGM) and arylamine-*N*-acetyltransferase (TBNAT), were selected as drug targets to reveal the mechanism underlying the antitubercular effect of fucoxanthin. The obtained results showed that fucoxanthin showed a clear bacteriostatic action against the all Mtb strains tested, with minimum inhibitory concentrations (MIC) ranging from 2.8 to 4.1 µM, along with a good degree of selectivity index (ranging from 6.1 to 8.9) based on cellular toxicity evaluation compared with standard drug isoniazid (INH). The potent inhibitory actions of fucoxanthin and standard uridine-5’-diphosphate against UGM were recorded to be 98.2% and 99.2%, respectively. TBNAT was potently inactivated by fucoxanthin (half maximal inhibitory concentration (IC_50_) = 4.8 µM; 99.1% inhibition) as compared to INH (IC_50_ = 5.9 µM; 97.4% inhibition). Further, molecular docking approaches were achieved to endorse and rationalize the biological findings along with envisaging structure-activity relationships. Since the clinical evidence of the last decade has confirmed the correlation between bacterial infections and autoimmune diseases, in this study we have discussed the linkage between infection with Mtb and autoimmune diseases based on previous clinical observations and animal studies. In conclusion, we propose that fucoxanthin could demonstrate great therapeutic value for the treatment of tuberculosis by acting on multiple targets through a bacteriostatic effect as well as by inhibiting UGM and TBNAT. Such outcomes may lead to avoiding or decreasing the susceptibility to autoimmune diseases associated with Mtb infection in a genetically susceptible host.

## 1. Introduction

*Mycobacterium tuberculosis* (Mtb) is a pathogenic microorganism that targets alveolar macrophages and induces tuberculosis in humans and animals [1]. Tuberculosis is one of the main global health challenges of all time. Although the current antitubercular drugs applied in the clinic have lessened mortality, Mtb resistance to these medications has become a global challenge facing public health [2,3]. Galactofuranose (Gal*f*) is a critical part of the arabinogalactan that connects the peptidoglycan layer and the mycolic acid layer in the cell wall of Mtb [4]. UDP-galactopyranose mutase (UGM) is a key enzyme involved in Gal*f* metabolism and catalyzes the interconversion of UDP-galactopyranose (UDP-Gal*p*) to UDP-galactofuranose (UDP-Gal*f*) [5]. Removal of the gene encoding for UGM in Mtb revealed that this enzyme is necessary for mycobacterial cell wall biosynthesis. Since Gal*f* and UGM are not detected in humans, UGM has been reported to be a vital target for therapeutic intervention [6,7]. Arylamine-*N*-acetyltransferase (NAT) is a cytosolic enzyme that catalyzes the transfer of the acetyl group from acetyl coenzyme A (AcCoA) to the free amino group of arylamines and hydrazines [8]. Mtb contains and expresses the gene encoding the NAT protein, and hence deleting the gene for arylamine-*N*-acetyltransferase from Mtb (TBNAT) leads to a decrease of mycobacterial cell wall lipid biosynthesis [9,10]. Therefore, TBNAT has been observed to be a crucial drug target for tuberculosis treatment [10]. The development of new antitubercular agents is imperative and is required to provide additional therapeutic alternatives. Over the past few decades, natural products, especially marine-derived metabolites, have gained considerable attention in drug discovery [11].

Fucoxanthin is a naturally occurring carotenoid and is extensively scattered in edible brown seaweeds and diatoms [12,13]. This molecule belongs to the group of xanthophylls and non-provitamin A carotenoids and contains unusual functional groups, including a unique allenic bond, nine unconjugated double bonds, a 5,6-monoepoxide moiety, and other oxygenic functional moieties [14,15]. While fucoxanthin remains a poorly studied marine metabolite, this substance has been reported to provide various health benefits, including antioxidant effects, anticancer properties, anti-obesity and anti-diabetic activities, anti-acetylcholinesterase properties, and good effects on the cardiovascular system through reducing cholesterol and triacylglycerol levels as well as lowering blood pressure and inflammatory processes [16,17,18,19,20,21]. In this report, we aimed to explore, for the first time, the antitubercular properties of fucoxanthin against clinical isolates of Mtb along with the inhibition activities against UGM and TBNAT. Besides, the cellular toxicity of fucoxanthin has been evaluated. The correlation between mycobacterial infection and autoimmune diseases has been also debated. 

## 2. Results and Discussion

### 2.1. Antimycobacterial and Cytotoxicity Characteristics

The antimicrobial activity of fucoxanthin and the standard drug isoniazid (INH) was assessed by microdilution assay against ten clinical isolates of Mtb along with a reference strain. As shown in Table 1, fucoxanthin exhibited a pronounced bacteriostatic effect on the all Mtb strains tested (expressed as minimum inhibitory concentration (MIC) values ranging from 2.8 to 4.1 µM) as compared to INH (MIC values ranging from 4.8 to 6.2 µM). 

Globally, the fast-rising frequency of infection with Mtb resulted in an extensive use of antibiotics, which in turn led to the development of drug resistance. Consequently, treatment failure has been established. Therefore, to defeat such complexities there is an inescapable necessity to find alternative sources that afford lower resistance and reduced adverse effects with the lowest possible cytotoxicity. Notably, the collected MICs for INH are in the line with the defined MIC breakpoints for INH (recorded for susceptible Mtb strains) as previously conveyed by the Clinical and Laboratory Standards Institute (CLSI) [22]. Although few reports have described the antimicrobial potency of fucoxanthin against various pathogenic bacteria except Mtb, we tried to rationalize our results with these studies [23,24,25,26]. However, we present a first investigation on the anti-Mtb activity of fucoxanthin. 

The fundamental principle of drug discovery and development is to design a drug that reaches its supposed target at a proper concentration and without cytotoxicity. Therefore, it is an essential process to assess the potential cytotoxicity of any drug that could interfere with its biological action [27,28]. Fucoxanthin and INH were tested for their possible cytotoxic impact on normal human fetal lung fibroblast cells. The half maximal inhibitory concentration (IC_50_) values for test molecules that are necessary to diminish the viability of test cells to 50% compared to a control (100% cell viability) are presented in Table 1. The results advocate that no cellular toxicity was manifested when cells treated with the test inhibitors, even at a high IC_50_ value of 25 µM. Thus, the safety index of fucoxanthin was authenticated by assessing the selectivity index (SI), where fucoxanthin had a cytotoxic effect on normal human fetal lung fibroblast cells at a concentration (>25 µM) higher than its MIC values. Our results are in line with a previously published study in which fucoxanthin only induced significant cytotoxicity against human epithelial cervical cancer cells with an IC_50_ value of 55 µM [29]. On the other hand, other studies confirmed the cytotoxic properties of fucoxanthin with a varied range of IC_50_ values [30,31,32,33]. The differences in obtained IC_50_ values could be assigned to the variation in test assays used, as well as different cell lines sources and experimental conditions used.

In autoimmunity research, it is currently recognized that susceptibility to autoimmune diseases is multifactorial and that the interplay between genetic and environmental factors triggers disease development [34]. Various genetic agents relate to susceptibility to autoimmune disease, and multiple environmental factors are responsible for cracking the tolerance to self-antigens in the susceptible host [35]. Infections with bacterial pathogens, which act as environmental activators, could induce or promote autoimmunity, resulting in clinical manifestations of autoimmune diseases in genetically susceptible individuals [36]. However, how infections are involved in disease inception or flare of many autoimmune diseases remains unknown [35]. Several significant review articles have comprehensively documented and clarified the association between infection with Mtb and susceptibility to autoimmune diseases based on in vivo and clinical studies [35,37,38,39,40]. Over the past few decades, many treatment strategies have been developed to manage tuberculosis infection, which in turn could pave the way to prevent or decrease the susceptibility to autoimmune diseases linked with this infection [37]. Unfortunately, the development of drug resistance has resulted in a failure of Mtb management. Therefore, new strategies based on natural-based medicine should be considered in large-scale research as an alternative means to conventional therapies. 

### 2.2. Anti-UGM Evaluation

This enzyme has been outlined to be critical macromolecule for the biosynthesis of the Mtb cell wall and hence considered an important drug target for tuberculosis therapy. Thus, we further examined whether the bacteriostatic effect of fucoxanthin against Mtb could be attributed to the inhibition of UGM. As presented in Table 2, fucoxanthin was tested in a UGM inhibition assay, where it efficiently inactivated the catalytic function of UGM (turnover 1.1% and inhibition 98.2%) compared with the standard inhibitor uridine-5’-diphosphate (UDP) (turnover 0.5% and inhibition 99.2%).

It has been proven that the growth of Mtb could be suppressed by inactivating UGM activity [41]. Hence, our results validated this observation, where the antimycobacterial efficacy of fucoxanthin was noted to be related to its inhibitory action against UGM. Since we here provide a first study on the anti-UGM activity of fucoxanthin and no prior studies had been conducted to reveal the inhibitory effects of carotenoids or terpenoids against UGM, we attempted to rationalize our results with a research report in which natural flavonoids showed potent inhibition properties against the UGM of Mtb [42].

### 2.3. Anti-TBNAT Evaluation

TBNAT plays a critical role in Mtb cell wall lipid biosynthesis and hence is appraised as a pivotal drug target for tuberculosis treatment. Consequently, we conducted anti-enzymatic assay against TBNAT to unveil the possible mechanism behind the anti-Mtb activity of fucoxanthin. The results demonstrated that fucoxanthin potently inhibited the activity of TBNAT (IC_50_ = 4.8 µM; 99.1% inhibition) compared with INH (IC_50_ = 5.9 µM; 97.4% inhibition) (Table 3). 

In this work, the inhibition activity of fucoxanthin against TBNAT is reported. Several publicized in vitro and in vivo studies were found to concur with our obtained results, where natural products were declared to have significant inhibitory effects on various types of NAT enzymes (microbial and human enzymes) [43,44,45,46].

### 2.4. In Silico Analyses

To further gain insights into the binding mode along with the molecular interactions between fucoxanthin and UGM (Mtb H_37_Rv; PDB ID: 4RPJ) and TBNAT (Mtb H_37_Rv; PDB ID: 4BGF), molecular docking analyses were accomplished. These studies were also conducted to validate and justify the outcomes of in vitro assays. Both enzymes were selected as valuable drug targets for the treatment of Mtb infection. 

#### Binding Modes and Molecular Interactions Between Fucoxanthin and UGM and TBNAT

**Figure 1:**
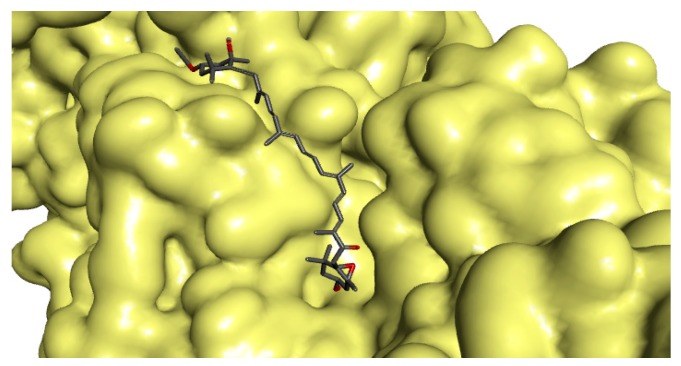
Protein–ligand illustration shows fucoxanthin fastens in the active site of the three-dimensional molecular surface of UDP-galactopyranose mutase (UGM). The docking score for fucoxanthin with the enzyme was found to be −8.5 kcal/mol (expressed as a binding affinity value).

**Figure 2:**
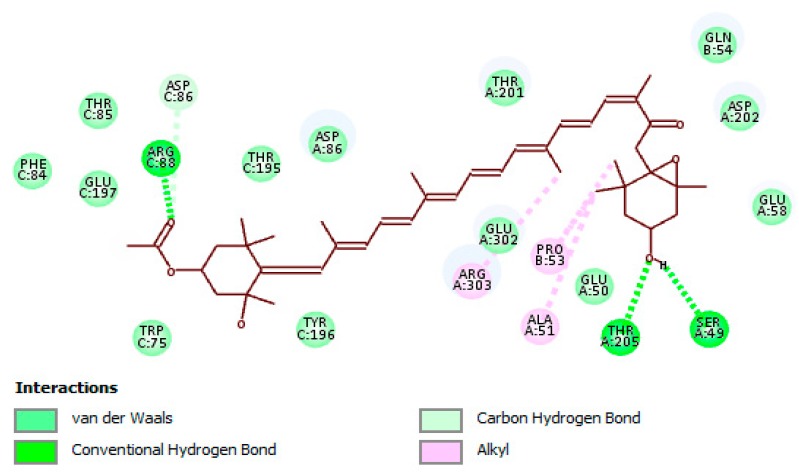
Binding mode and molecular interaction of fucoxanthin with UDP-galactopyranose mutase (UGM). Important interactions with amino acid residues of the active site of the enzyme are depicted. Key functional groups of fucoxanthin that are responsible for creating the molecular interactions with the active site are displayed.

The docking results revealed that fucoxanthin effectively bound to the active site of UGM (Figure 1). The molecular interaction between fucoxanthin and UGM was formed by crucial connections such as hydrogen bonding, carbon–hydrogen bonding, hydrophobic, and van der Waals interactions with the amino acid residues found in UGM active site, along with the functional groups of fucoxanthin that are accountable for the interactions (Figure 2). All detected amino acid residues were previously suggested to be essential for enzyme suppression [47]. The remarked binding mode of fucoxanthin with UGM active site indicates that this molecule competes with substrate for dominating the active site and accordingly we may suggest fucoxanthin as a competitive inhibitor of UGM.

**Figure 3:**
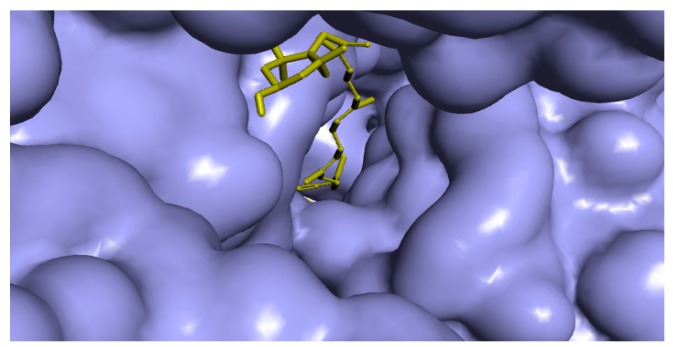
Protein–ligand depiction demonstrates fucoxanthin fastens in the active site of the three-dimensional molecular surface of arylamine-*N*-acetyltransferase from *M. tuberculosis* (TBNAT). The docking score for fucoxanthin with the enzyme was found to be −7.9 kcal/mol (presented as a binding affinity value).

**Figure 4:**
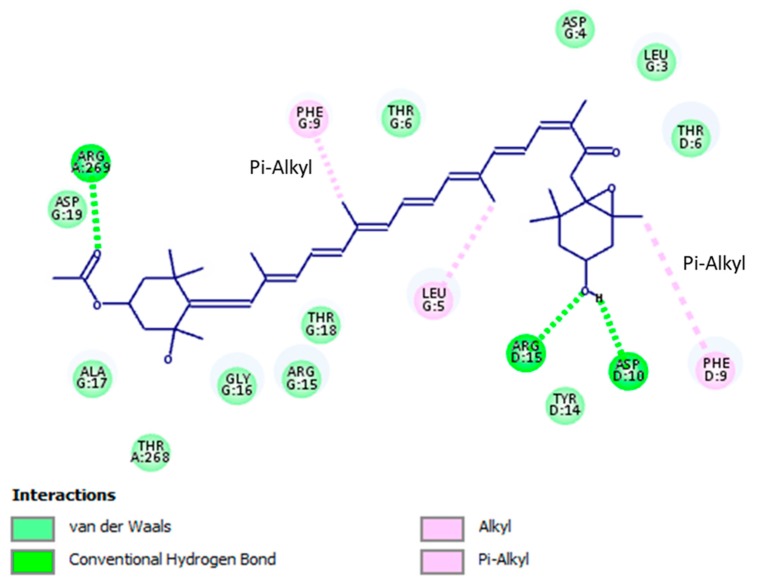
Binding mode and molecular interaction of fucoxanthin with arylamine-*N*-acetyltransferase (TBNAT). Imperative interactions with amino acid residues of the active site of the enzyme are described, where crucial functional groups of fucoxanthin that are accountable for setting up the molecular interactions with the active site are displayed.

The docking outcomes of fucoxanthin-TBNAT complex displayed noticeable binding mode (Figure 3). This binding mode has established significant molecular interactions via forming substantial contacts such as hydrogen bonding, hydrophobic, and van der Waals interactions with amino acid residues of TBNAT active site (Figure 4). Besides, the functional groups of fucoxanthin that perform a vital role in setting up the detected interactions were perceived. All observed amino acid residues of TBNAT active site were previously proposed to be vital for inhibiting TBNAT activity [48,49]. Based on the above-mentioned findings, we may propose fucoxanthin as a competitive inhibitor of TBNAT, where this compound remarkably fitted in the active site and hence struggled with the substrate to reside in the active site. 

Overall, it is important to declare that the results acquired from molecular docking analyses are in accordance with the data of in vitro tests, which, in turn, justify the inhibitory characteristics of fucoxanthin against UGM and TBNAT.

## 3. Materials and Methods 

### 3.1. Antimicrobial Activity

#### 3.1.1. Mycobacterial Strains and Culture Conditions 

Ten clinical isolates of Mtb (A, B, C, D, E, F, G, H, I, and J; isolated from patients infected with Mtb) were kindly received from Motol University Hospital (MUH) in Prague, Czech Republic, whereas the reference bacterial strain of Mtb (H_37_Rv; CNCTC My 331-88: ATCC 27294) was gained from the Czech National Collection of Type Cultures (CNCTC), National Institute of Public Health, Prague, Czech Republic. The identification of all clinical isolates was ascertained employing biochemical and molecular techniques of the certified guideline of Clinical and Laboratory Standards Institute (CLSI) [50]. Fucoxanthin and isoniazid (INH; a standard antitubercular medication) were purchased from Sigma-Aldrich, Berlin, Germany. All test Mtb strains were observed to be susceptible to INH, and then were cultured and grown following the guideline of the CLSI [22].

#### 3.1.2. Assessment of Minimum Inhibitory Concentration (MIC)

Minimum inhibitory concentration of fucoxanthin and INH was assessed by microdilution assay as previously described in CLSI guideline [22]. The negative controls were assigned to be dimethyl sulfoxide (DMSO) and the broth. Here, 1% DMSO was employed to dissolve and dilute the test compounds and further mixed with broth (25 µL of DMSO solution in 5 mL of broth). The used 1% of DMSO had no effect on the growth of Mtb. Final concentrations of test substances in wells ranged from 2.8 to 6.2 µM. After 24 h of incubation, the results were registered and represented as minimal inhibitory concentration (MIC) at a micromolar scale that impeded the blue to pink color change.

### 3.2. Cellular Toxicity Analysis

#### 3.2.1. Cell Lines and Culture Requirements

Normal human fetal lung fibroblast cells (MRC-5; MUH, Prague, Czech Republic) were cultured in Minimum Essential Eagle Medium MEM (Sigma-Aldrich, Berlin, Germany) supplemented with 10% fetal bovine serum (PAA Laboratories GmbH, Pasching, Autria), 2 mM _L_-glutamine solution (Sigma-Aldrich, Berlin, Germany), and 1% non-essential amino acid solution (Sigma-Aldrich, Berlin, Germany), in a humidified state with 5% CO_2_ at 37 °C. Subsequently, for subculture the cells were collected after treatment with trypsin/EDTA (Sigma-Aldrich, Berlin, Germany) at 37 °C [51].

#### 3.2.2. Cytotoxicity Assessment 

The potential cytotoxicity of fucoxanthin and INH was assessed using a CellTiter‒96 assay. This assay is based on the reduction of tetrazolium dye MTS in living cells to formazan, which is then ascertained colorimetrically following the hitherto reported procedure [51]. The MRC-5 cells treated with the test compounds were applied as investigational groups, whereas untreated MRC-5 cells acted as control groups. The absorbance of test samples was detected at 490 nm and the inhibitory curves were assembled for each compound, using incubation concentrations vs. percentage of absorbance relative to untreated control. The half maximal inhibitory concentration (IC_50_) was ascertained by a nonlinear regression analysis of the inhibitory curves. The statistical analyses were aided by PRISM software (GraphPad Software, Inc., La Jolla, CA, USA; version 8.0). 

### 3.3. UGM Assay

#### 3.3.1. UGM Expression and Purification System

The standard strain Mtb (H_37_Rv CNCTC My 331-88; ATCC 27,294 obtained from the Czech National Collection of Type Cultures, National Institute of Public Health, Prague, Czech Republic) was used as a source for the expression and purification of the enzyme. The expression and purification system along with the required experimental conditions were followed as previously detailed [52]. The quantitation of the obtained enzyme was assessed as earlier described in detail [53].

#### 3.3.2. UGM Activity

UGM activity was accomplished as previously specified [54,55]. Fucoxanthin and the standard UGM inhibitor uridine-5’-diphosphate (UDP; Sigma Chemical Co., St Louis, MO, USA) were prepared in DMSO (DMSO 2% (v/v) was used in all performed reactions). Concisely, UGM (25 µg/mL) prepared in a buffer (100 mM of 3-(N-morpholino) propanesulfonic acid (MOPS); pH = 8.0) was pre-incubated with Na_2_S_2_O_3_ (20 mM) on ice for 1 min. Further, the solution mixture was incubated with the test inhibitors (20 mM) followed by adding the substrate UDP-Gal*f* (60 µM; MUH, Prague, Czech Republic) at 25 °C. Subsequently, the reactions were stopped at various times by adding ice-cold HCl and directly subjected to quick freezing in liquid nitrogen. The activity of UGM in the presence of 2% (v/v) DMSO was considered a control. A high performance liquid chromatography (HPLC) system (Agilent 1100 series) was applied to monitor UGM activity, where all instrumental setup and operational requirements were tracked according to the detailed procedures [54,55]. The degree of conversion was measured throughout the comparison of the integration of substrate and product peaks.

### 3.4. TBNAT Assay

#### 3.4.1. TBNAT Expression and Purification System

A comprehensive system for protein expression and purification was applied to produce TBNAT in a form of recombinant protein utilizing a detailed protocol described by Abuhammad et al. [56]. After expression and purification of TBNAT, the enzyme was stocked for additional use at −80 °C in Tris-HCl (20 mM; pH = 8) blended with dithiothreitol (1 mM) and glycerol (5%). 

#### 3.4.2. TBNAT Activity

Microplate photometer-based assay was subjected to determine TBNAT-catalyzed reaction with slight refinement [57]. TBNAT activity was detected by monitoring the rate of hydrolysis of acetyl coenzyme A (AcCoA) through detection with 5,5′-dithio-bis(2-nitrobenzoic acid) (DTNB), and the absorbance was recorded at 405 nm (Tecan Sunrise Plate Reader, Männedorf, Switzerland). To sum up, the test molecules (fucoxanthin and standard INH) were prepared and dissolved in DMSO and all reactions were processed in the presence of DMSO (2%; v/v). TBNAT (170 ng; prepared in 20 mM Tris-HCl (pH = 8) mixed with dithiothreitol (1 mM) and 5% glycerol) was incubated with test compounds (5 µL at final concentrations ranging from 10 to 20 µM) for 15 min at 25 °C. Further, 15 µL of a substrate hydralazine (30 µM; Sigma-Aldrich, Berlin, Germany) and 12 µL of acetyl CoA (30 µM) were blended with the obtained mixture solution. Subsequently, the reaction was stopped by utilizing 25 µL of DTNB (processed in guanidine-HCl (6.4 M) and Tris-HCl (100 mM) with pH = 7.3) after 10 min at 25 °C and the enzyme activity was achieved as an end-point readout analysis. The TBNAT-catalyzed reaction (no inhibition) was assigned as a control. The % inhibition was ascertained as the ratio of enzyme activity (expressed as the rate of CoA formation with test molecules) to the activity of the control without inhibition. The inhibitory curves which were obtained by non-linear fitting of the % inhibition and the logarithmic concentration of the inhibitor versus the response were used to assess IC_50_ values. 

### 3.5. In Silico Investigation

The PyRx docking tool fixed with Autodock VINA software (version 0.8, The Scripps Research Institute, La Jolla, CA, USA) was utilized for conducting the molecular docking analyses, whereas the RCSB Protein Data Bank (www.rcsb.org) was employed for retrieving the three-dimensional (3D) crystal structure of UDP-galactopyranose mutase from Mtb docked with UDP (UGM; PDB ID: 4RPJ), the 3D-crystal structure of arylamine-*N*-acetyltransferase from Mtb (TBNAT; PDB ID: 4BGF), and the 3D-structure of fucoxanthin (SDF ID file: A86). The docking results were verified by removing the ligand (UDP) from the PDB (PDB ID: 4RPJ) structure and re-docked back into the crystal structure of the enzyme with docking score −6.2 kcal/mol. The docking analyses were studied based on binding affinity values of the obtained enzyme-ligand complexes (kcal/mol) along with hydrogen bonding, hydrophobic, and electrostatic interactions. The docking settings, preparation of PDBQT files for the enzymes and ligand, calculations, the protonation state, and the total charges were ascertained as previously detailed [58]. All docking results were graphically displayed using Discovery studio visualizer version v19.1.0.18287 (BIOVIA, San Diego, CA, USA) [59].

## 4. Conclusions

In this study, the role of fucoxanthin as an antitubercular molecule has been explored. Fucoxanthin unveiled effective anti-Mtb property with MIC values ranging from 2.8 to 4.1 µM and SI (ranging from 6.1 to 8.9). We also determined the remarked anti-enzymatic properties of fucoxanthin against UGM and TBNAT as crucial drug targets implicated in Mtb cell wall biosynthesis. Anti-Mtb mechanism studies specified that the mechanism by which fucoxanthin-induced anti-Mtb activity could be related to its inhibitory action against UGM and TBNAT. Additionally, the safety index of fucoxanthin was determined. Molecular docking studies validated and rationalized the biological results obtained from in vitro assays. To sum up, fucoxanthin could be used as a candidate for the development of antimycobacterial agents, and hence could open new gates for overwhelming the problem of drug-resistant strains. Moreover, in-depth in vivo investigations and clinical trials should be further considered along with pharmacokinetic and pharmacodynamic studies. Integrated and comprehensive studies are also required to reveal the mechanism underlying the antitubercular activity of fucoxanthin. It is very important to highlight that our obtained results could lead to averting or diminishing the susceptibility to autoimmune diseases linked with Mtb infection in the genetically susceptible host. 

## Figures and Tables

**Table 1:** Bacteriostatic effect of fucoxanthin and isoniazid on *Mycobacterium tuberculosis* strains and cytotoxicity evaluation.

Mycobacterial Strains	MIC (µM)		Cytotoxicity (IC_50_; µM) for Fucoxanthin and INH	SI	
	Fucoxanthin	INH		Fucoxanthin	INH
Mtb ^a^	4.1	6.2	>25	>6.1	>4.0
Mtb-A ^b^	3.9	5.8	>25	>6.4	>4.3
Mtb-B ^b^	3.9	5.2	>25	>6.4	>4.8
Mtb-C ^b^	3.8	5.3	>25	>6.6	>4.7
Mtb-D ^b^	3.5	5.5	>25	>7.1	>4.5
Mtb-E ^b^	3.6	5.5	>25	>6.9	>4.5
Mtb-F ^b^	2.9	4.8	>25	>8.6	>5.2
Mtb-G ^b^	2.9	4.9	>25	>8.6	>5.1
Mtb-H ^b^	3.0	5.1	>25	>8.3	>4.9
Mtb-I ^b^	2.8	4.8	>25	>8.9	>5.2
Mtb-J ^b^	3.1	5.2	>25	>8.1	>4.8

The displayed values demonstrate the average of three independent measurements conducted in triplicate. ^a^ Mtb: *Mycobacterium tuberculosis* (standard strain; H_37_Rv CNCTC My 331-88/ATCC 27294); ^b^ Mtb: Clinical isolates of *Mycobacterium tuberculosis*; MIC: minimum inhibitory concentration; INH: isoniazid; IC_50_: half maximal inhibitory concentration (expressed as toxicological value); SI: selectivity index expressed as the ratio IC_50_/MIC. The statistical analyses were aided by PRISM software (GraphPad Software, Inc., La Jolla, CA, USA; version 8.0).

**Table 2:** Anti-enzymatic properties of fucoxanthin and UDP against UGM.

Inhibitors	Turnover ^a^ (%)	Inhibition ^b^ (%)
Fucoxanthin	1.1 ± 0.2	98.2
UDP	0.5 ± 0.4	99.2
No inhibition	61.3 ± 0.7	Nd

PRISM software (GraphPad Software, Inc., La Jolla, CA, USA; version 8.0) was employed for performing statistical analyses. The data are displayed as the mean ± standard deviation (SD); each measurement was conducted in triplicate. ^a^ % Turnover was ascertained by integration of the peak of the substrate and the peak of the product. ^b^ % Inhibition was assessed from the % turnover of the decreased reaction (in the presence of inhibitors) compared to the reaction in the absence of inhibition (no inhibition). Nd: not determined; UDP: uridine-5’-diphosphate; UGM: UDP-galactopyranose mutase.

**Table 3:** Anti-enzymatic actions of fucoxanthin and INH against TBNAT.

Inhibitors	Inhibition (%)	IC_50_ (µM)
Fucoxanthin	99.1 ± 0.6	4.8 ± 0.4
INH	97.4 ± 0.4	5.9 ± 0.2

Statistical analyses were performed by PRISM software (GraphPad Software, Inc., La Jolla, CA, USA; version 8.0). The results are shown as the mean ± standard deviation (SD) of three measurements performed in triplicate. IC_50_: half maximal inhibitory concentration; INH, isoniazid; TBNAT: *Mycobacterium tuberculosis* arylamine-*N*-acetyltransferase.
